# Supplementary material for: MetaQTL: a package of new computational methods for the meta-analysis of QTL mapping experiments
Source: BMC Bioinformatics. 2007 Feb 8;8:49. doi: 10.1186/1471-2105-8-49 (PMC1808479; doi:10.1186/1471-2105-8-49)
Supplement: Additional File 7 — MetaQTL Package : jar file and tutorial. This Zip archive contains both the MetaQTL JAR file and the files of the tutorial. [file 1471-2105-8-49-S7.zip › org.inra.metaqtl/doc/org/thalia/bio/entity/OntologyTerm.html]

OntologyTerm


|  |  |  |
| --- | --- | --- |
| **Overview** \| **Package** \| **Class** \| **Use** \| **Tree** \| **Deprecated** \| **Index** \| **Help** | |  |
| **PREV CLASS**   **NEXT CLASS** | **FRAMES**    **NO FRAMES**     **All Classes** |  |
| SUMMARY: NESTED \| FIELD \| CONSTR \| METHOD | DETAIL: FIELD \| CONSTR \| METHOD |  |


---


## org.thalia.bio.entity Class OntologyTerm

```
java.lang.Object
  org.thalia.bio.entity.BioEntity
      org.thalia.bio.entity.OntologyTerm
```

**All Implemented Interfaces:**: IBioAdaptable, IBioEntity, IBioOntologyTerm

---

``` public class OntologyTerm extends BioEntity implements IBioOntologyTerm ```

---

**Field Summary**

**Fields inherited from class org.thalia.bio.entity.BioEntity**

`name, parent, properties`


**Constructor Summary**

`OntologyTerm()`


| **Method Summary** | |
| --- | --- |
| `IBioAdapter` | `getBioAdapter()` |
| `IBioOntologyTerm[]` | `getChildrenTerm()`             Returns the children if the term has chilren, null otherwise. |
| `long` | `getID()`             Returns the id if the terms. |
| `IBioOntologyTerm` | `getParentTerm()`             Returns the parent of the term. |
| `int` | `getType()`             There are 2 main class of entities. |
| `boolean` | `hasChildren()`             Returns true if the term has children, false otherwise. |
| `void` | `setChildren(OntologyTerm[] children)`             Sets the children of the term. |
| `void` | `setID(long id)`             Sets the indentifient of the term. |
| `void` | `setParentTerm(IBioOntologyTerm parentTerm)`             Sets the parent of the term. |

**Methods inherited from class org.thalia.bio.entity.BioEntity**

`getName, getParent, getProperties, newBioEntity, setName, setProperties`

**Methods inherited from class java.lang.Object**

`clone, equals, finalize, getClass, hashCode, notify, notifyAll, toString, wait, wait, wait`

**Methods inherited from interface org.thalia.bio.IBioEntity**

`getName, getParent, getProperties, setName, setProperties`

**Constructor Detail**

### OntologyTerm

```
public OntologyTerm()
```


**Method Detail**

### getID

```
public long getID()
```

:   **Description copied from interface: `IBioOntologyTerm`**
:   Returns the id if the terms.

    :   **Specified by:**: `getID` in interface `IBioOntologyTerm`
    :   **Returns:**

---


### getChildrenTerm

```
public IBioOntologyTerm[] getChildrenTerm()
```

:   **Description copied from interface: `IBioOntologyTerm`**
:   Returns the children if the term has chilren,
    null otherwise.

    :   **Specified by:**: `getChildrenTerm` in interface `IBioOntologyTerm`
    :   **Returns:**

---


### hasChildren

```
public boolean hasChildren()
```

:   **Description copied from interface: `IBioOntologyTerm`**
:   Returns true if the term has children, false
    otherwise.

    :   **Specified by:**: `hasChildren` in interface `IBioOntologyTerm`
    :   **Returns:**

---


### getType

```
public int getType()
```

:   **Description copied from interface: `IBioEntity`**
:   There are 2 main class of entities. The first one deals with population
    biological entity, i.e population itself and individuals. The second class
    is a representation of microscopic biological entity from genome container
    to alleles.

    :   **Specified by:**: `getType` in interface `IBioEntity` **Specified by:**: `getType` in class `BioEntity`

---


### getBioAdapter

```
public IBioAdapter getBioAdapter()
```

:   **Specified by:**: `getBioAdapter` in interface `IBioAdaptable`

---


### setID

```
public void setID(long id)
```

:   Sets the indentifient of the term.

    :   **Parameters:**: `id` - the new identifient of the term.

---


### setChildren

```
public void setChildren(OntologyTerm[] children)
```

:   Sets the children of the term.

    :   **Parameters:**: `children` - the children of the term.

---


### getParentTerm

```
public IBioOntologyTerm getParentTerm()
```

:   **Description copied from interface: `IBioOntologyTerm`**
:   Returns the parent of the term.

    :   **Specified by:**: `getParentTerm` in interface `IBioOntologyTerm`
    :   **Returns:**

---


### setParentTerm

```
public void setParentTerm(IBioOntologyTerm parentTerm)
```

:   Sets the parent of the term.

    :   **Parameters:**: `parent` -


---


|  |  |  |
| --- | --- | --- |
| **Overview** \| **Package** \| **Class** \| **Use** \| **Tree** \| **Deprecated** \| **Index** \| **Help** | |  |
| **PREV CLASS**   **NEXT CLASS** | **FRAMES**    **NO FRAMES**     **All Classes** |  |
| SUMMARY: NESTED \| FIELD \| CONSTR \| METHOD | DETAIL: FIELD \| CONSTR \| METHOD |  |


---
